# Obesity and craniopharyngioma

**DOI:** 10.1186/1824-7288-37-38

**Published:** 2011-08-16

**Authors:** Lorenzo Iughetti, Patrizia Bruzzi

**Affiliations:** 1 Department of Paediatrics, University of Modena & Reggio Emilia, Modena, Italy

**Keywords:** Obesity, Craniopharyngioma, Children, Hypothalamic-pituitary, axis

## Abstract

An epidemic of pediatric obesity has occurred across the world in recent years. There are subgroups within the population at high-risk of becoming obese and especially of having experience of precocious cardiovascular and metabolic co-morbidities of obesity. One of these subgroups comprises patients treated for childhood cancers and namely survivors of craniopharyngioma. The high incidence of obesity in this group makes these patients an important disease model to better understand the metabolic disturbances and the mechanisms of weight gain among cancer survivors. The hypothalamic-pituitary axis damage secondary to cancer therapies or to primary tumor location affect long-term outcomes. Nevertheless, the aetiology of obesity in craniopharyngioma is not yet fully understood. The present review has the aim of summarizing the published data and examining the most accepted mechanisms and main predisposing factors related to weight gain in this particular population.

## Introduction

As results of advances in treatment, almost 80% of children and adolescents who receive a diagnosis of cancer become long-term survivors. In the United States, there are approximately 270.000 survivors of pediatric cancer reaching the amount of about 1 every 640 adults between the ages of 20 and 39 years [1]. The improved survival rates have resulted in increased attention on late side-effects. They can include second malignancies, cardiovascular abnormalities, pulmonary complications, endocrine consequences and obesity. In the Childhood Cancer Survivor Study, the survivor population is considered a high-risk population. Thirty years after a diagnosis of cancer, almost three fourths of survivors have a chronic health condition, more than 40% have a serious health problem and one third have multiple conditions [2]. Some of these adverse health effects may be modifiable and, therefore, the long-term monitoring of survivors has became an important part of their overall health care and the identification of treatment and patient factors that contribute to them is now crucial.

Obesity is a well-recognized complication of tumors localized in the hypothalamic-pituitary region. In childhood, craniopharyngioma is the most common neoplasm of the hypothalamic-pituitary area, accounting for approximately 80% of tumors in this location and represents 5-15% of intracranial tumors [3]. A Childhood Cancer Registry estimates an incidence of craniopharyngioma of 1.4 cases per million children per year in Italy [4]. Similar data are provided by other registries in Western countries, while higher rates have been observed in Asia and Africa. Craniopharyngioma has a bimodal age distribution with a peak between 5 and 14 years of age, and a second one in adults older than 65 years. Even if it is not truly malignant, it is locally invasive and grows slowly in the suprasellar region. During its growth, craniopharyngioma could invade surrounding tissue including the hypothalamus and optic chiasm. Consequently, even if survival has dramatically improved in the last years (overall 10-years survival rate: 91-98%), this attachment makes the complete excision of the tumor difficult and the affected children may be visually impaired and often develop multiple endocrine deficiencies [5]. Management of craniopharyngioma is therefore still complex and controversial. Aiming the preservation of the hypothalamic function, a complete resection of the tumor is the treatment of choice only in patients with favorable localization. When the tumor localization is unfavorable, a limited resection followed by local irradiation is recommended [6].

In brain tumors, the location of the primary tumors might be one of the principal factors affecting body weight and composition. The hypothalamic obesity is defined as a rapid, unrelenting and intractable weight gain, not responsive to diet and exercise and usually associated with others symptoms, including headache, impaired vision, increased somnolence and behavioral disturbances, especially severe hyperphagia and abnormal food seeking behaviors [7].

Hypothalamic involvement can derive by infiltration of the tumor itself. In such cases, weight gain could be a sign of the disease leading, together with other symptoms, to the diagnosis of craniopharyngioma. In a large study of 63 children, 17% had a history of significant weight gain in the 3.5 years before the diagnosis of craniopharyngioma [8].

After tumor resection, survivors of childhood craniopharingioma experienced an about 50% increased rate of obesity [9-11]. In a study conducted in the St. Jude Children's Research Hospital, 65% of 55 patients treated for craniopharyngioma during childhood (about 90% surgery plus radiotherapy) were found overweight or obese after 7.6 years (range 5-21.3 years) from diagnosis [12]. Muller showed that the extension of the tumor to the hypothalamus was found in 76% of obese and 96% of severely obese patients compared to 33% of normal weight survivors [13]. Following surgery, mean Body-Mass Index (BMI) Standard Deviation Score (SDS) increased from 0.0 to 2.7 at 6 months and to 2.4 at 1 year [8]. The increase of BMI correlates with the severity of hypothalamic damage evaluated both on preoperative [9,14] and postoperative imaging [15,16].

In craniopharyngioma, a distinctive pattern of weight gain has already emerged. Children who developed hypothalamic obesity have a significant and rapid BMI increase over the first 6 months after treatment, followed by stabilization, with no subsequent loss of weight [15]. That is the reason why an early and rapid postoperative weight gain seems to be a significant predictive factor for severe long-term obesity [10,17]. In addition, patients who remained at normal weight during follow-up after diagnosis of childhood craniopharyngioma presented a lower BMI SDS at the time of the diagnosis [9,10]. Therefore, an increased BMI at diagnosis of craniopharyngioma is reported as another significant risk factor predictive of the development of severe obesity [18,19]. It could derived from the presence of a hypothalamic dysfunction prior the diagnosis of craniopharyngioma, but it could also prove the presence of an unhealthy lifestyle, already existing independently of the subsequent diagnosis of craniopharyngioma.

## Etiology of obesity in Craniopharyngioma

### Alteration of the hypothalamic signal pathway

In craniopharyngioma weight gain can principally occur from the disruption of the normal homeostatic function of the hypothalamic centers responsible for controlling satiety and hunger and regulating energy balance.

In physiological conditions, the hypothalamic arcuate nucleus (ARC), located in median eminence, where brain barrier is freely permeable, senses nutrient and hormonal signals from the periphery and is the primary site of two sets of neurons that form part of the central melanocortin system, a key regulator of energy balance. These two neuronal populations express both anorectic [proopiomelanocortin (POMC)-product alpha-melanocyte-stimulating hormone (α-MSH)] and orexigenic [agouti-related protein and neuropeptide Y (AGRP/NPY)] peptides. Both leptin, an adipose tissue-related cytokine, and insulin stimulate arcuate expression of POMC and suppress AGRP/MPY expression [20,21]. Disruption of responses to leptin and insulin from hypothalamic damage has been proposed as a possible mechanism contributing to hypothalamic obesity [7]. A disturbed feedback mechanism from the hypothalamic leptin receptors to the adipose tissue can be assumed, including a loss of leptin feedback inhibition on neuropeptide Y and appetite. Roth, evaluating serum leptin levels in 14 patients after neurosurgical treatment for craniopharyngioma, suggested that the hypothalamic structures might be insensitive to endogenous leptin. Normal controls had a positive correlation between leptin levels and BMI. Significantly elevated leptin levels with respect to BMI were found in 11 craniopharyngioma patients who had been affected by a suprasellar tumour, whereas 3 patients with an intrasellar tumor had lower, almost normal serum leptin levels [22]. It has been also showed leptin concentrations in serum of patients with craniopharyngioma increased over proportionally when patients developed obesity [23].

Animal studies suggest that hypothalamic obesity can also result from increased parasympathetic and decreased sympathetic activity [24]. In humans, an autonomic imbalance has been already confirmed [25]. Patients with a history of hypothalamic craniopharyngioma resection and high BMI values had an impaired counter regulatory response to hypoglycemia with significantly lower levels of plasma epinephrine [26]. Lesions of the ventromedial nuclei may lead to disinhibited or increased vagal tone resulting in hyperinsulinemia.

In fact, patients have an increased insulin secretion compared with BMI-matched controls and the circulating insulin levels increase with developing peripheral resistance to insulin action [27].

In 2009 Trivin et al evaluated the pre-surgery relationship between the degree of the hypothalamic involvement of craniopharyngioma patients on magnetic resonance imaging and insulin resistance, as evaluated by the homeostasis model insulin resistance index (HOMA). Despite having similar BMI, patients with severe hypothalamic involvement had higher glucose, insulin and HOMA before surgery than patients without hypothalamic involvement or only with hypothalamic compression [28]. Moreover, the successful results of a pharmacological trial have been considered an indirect proof of role of insulin hypersecretion due to β-cell dysfunction in hypothalamic obesity [29]. In this trial, 18 obese subjects, with a previous history of childhood craniopharyngioma received octreotide or placebo for 6 months. Treated patients experienced improved insulin response to glucose, weight loss or lack of weight gain, appetite suppression and reduced energy intake, increased reported physical activity and decreased leptin levels.

Furthermore, in craniopharyngioma survivors it has been identified a difference in insulin sensitivity that was found lower if analyzed by Frequently Sampled Intravenous Glucose Tolerance Test (FSIGT) rather than by Oral Glucose Tolerance Test (OGTT) [27]. This different response may indicate the mediation of factors such as autonomic innervation and gastrointestinal tract hormones in the pathogenetic process. For instance, the insulin secretagogue glucagon-like peptide 1 (GLP-1) is released from the enteroendocrine L cells in the distal intestine in response to glucose ingestion and leads to enhanced insulin secretion. Exaggerated GLP-1 secretion in response to an oral glucose load in individuals treated for craniopharyngioma might link the findings of insulin secretion and autonomic dysregulation. These are precocious data and further studies are required to investigate the hypothesis.

Ghrelin is a hormone produced mainly by P/D1 cells lining the fundus of the human stomach and epsilon cells of the pancreas [30]. It increases food intake and fat mass by an action exerted at the level of the ARC stimulating the orexigenic NPY neurons. The hyperghrelinemia observed in Prader-Willi Syndrome has encouraged investigation into whether elevated ghrelin levels are also present in hypothalamic obesity due to acquired structural damage, as in craniopharyngioma. Nevertheless, data supporting a role for hyperghrelinemia in the pathogenesis of hypothalamic obesity are still lacking [7,31]. Goldstone et al found that fasting plasma ghrelin in obese patients with craniopharyngioma was significantly lower than that of patients with Prader-Willi Syndrome and did not differ from levels found in control patients with common obesity [32]. Likewise, Kanumakala et al found no difference in fasting total ghrelin levels between patients with hypothalamic obesity and controls with common diet- induced obesity [33]. Recently, the dynamic response of ghrelin to oral glucose tolerance test was studied in 15 patients with hypothalamic obesity and craniopharyngioma and in 15 BMI-matched controls. From 0-30 minutes after stimulation, ghrelin decreased and insulin increased more in patients compared with controls. This delayed ghrelin suppression may contribute to obesity in obese craniopharyngioma patients, but further studies are necessary to better understand the meaning of this finding [34].

### Lifestyle

Even if impaired satiety and hyperphagia, caused by disruption of the hypothalamic nuclei, have a central role in the onset of hypothalamic obesity, reduced physical activity, rather than increased total energy intake, was found to be a major etiologic factor in the development of obesity in survivors of childhood craniopharyngioma [31]. Supporting data derived from the study of Harz and colleagues. In fact, they demonstrated that the caloric intake of patients with hypothalamic obesity from craniopharyngioma, when determined by a validated food diary, did not exceed that of control patients with common obesity, but their movement activity, assessed by accelerometer, was reduced both in the ambulatory setting and in a clinically monitored weight loss environment [35]. Moreover, Müller in 2002 reported an increased daytime sleepiness and reduced nocturnal melatonin levels in patients with childhood craniopharyngioma supporting the hypothesis that in these patients physical activity might be decreased due to as yet unknown neuroendocrine disorders [36]. There are several conditions may responsible of the reduction of energy expenditure in these patients. Hypopituitarism has to be considered one of these causes. Furthermore, the tumor or its treatment may result in specific neurological or behavioral problems affecting physical activity [37]. Functional capacity and its relation with weight gain have been evaluated in 212 patients with childhood craniopharyngioma. Patients with hypothalamic involvement presented higher BMI SDS at the time of diagnosis and at latest follow-up evaluation and lower functional capacity when compared with patients without hypothalamic involvement [17].

### Treatment modalities

Any form of hypothalamic damage, whether due to tumor, surgery or radiotherapy, is a regional-specific primary risk factor for the development of obesity. In a longitudinal retrospective BMI-analysis (6.2 years post-diagnosis) in 90 childhood craniopharyngioma patients, survivors with hypothalamic involvement had higher BMI at time of diagnosis and at annual follow-up intervals [9]. At latest follow-up, BMI SD was 5.1 in survivors with hypothalamic involvement versus 0.4 in those without hypothalamic involvement. Only 10% of survivors with hypothalamic involvement preserved normal weight (versus 69% of survivors without hypothalamic involvement), independent of the type of treatment. A progression of the hypothalamus damage, determined by different modalities of treatment, could deteriorate the weight gain. There are criticized evidences that an aggressive surgical intervention [13], a lower rate of gross total tumor resection during surgery [9] and multiple operations due to tumor recurrence [37] could worsen the degree of obesity [38]. In addition, in an elegant study of 148 survivors of childhood brain tumors, Lustig sustained that survivors who received cranial radiotherapy ≥ 51 Gy, especially if administered hypothalamic, had greater BMI increase compared to those who received less [18]. The multicenter Childhood Cancer Survivor Study (CCSS) showed that treatment with surgery and radiotherapy increased the risk of obesity in female survivors about 3-fold compared with surgery alone and documented the presence of a dose-response relation between hypothalamic radiation and obesity among females [39].

In craniopharyngioma, high-dose glucocorticoid treatment is usually proposed at presentation with the aim to ameliorate the symptoms of raised intracranial pressure or after surgery to limit the postoperative edema. In 2003 a retrospective analysis of 60 patients with childhood craniopharyngioma inquired whether dose and duration of perioperative dexamethasone therapy had influence on short-term post-operative weight gain and long-term development of severe obesity. Whereas cumulative dexamethasone doses positively correlated with weight gain during the first year following surgery, long-term development of severe obesity was not influenced by dose and duration of perioperative dexamethasone treatment [40].

### Hypopituitarism

The geography of the tumors as well as surgery and radiotherapy cause multiple pituitary hormone deficiencies. At diagnosis 71% of patients with craniopharyngioma have symptoms suggesting the presence of an endocrinopathy. Among them, 15% presented rapid and excessive weight gain. After a mean of 7 years from the initial surgery, multiple endocrinopathies are almost universal and 75% of children have panhypopituitarism [41]. Several hormone deficiencies could contribute to excess weight gain [18]. Although the adverse effects of radiotherapy are dependent on dose, fractionation, age at irradiation, time from irradiation and site, there is evidence that growth hormone (GH) is the most sensitive to these effects [42]. The irradiation of the hypothalamic-pituitary axis with 30 Gray (Gy) or more in fractions provoke growth hormone deficiency (GHD) with a risk of 60-100% within 2-5 years [43].

Evidences relate GHD to abnormal body composition, including decreased lean body mass and an increased percentage of body fat, especially around the waist, and to abnormal lipoprotein metabolism, increased peripheral insulin resistance and impaired glucose tolerance [44-46]. Moreover children who undergo cranial radiation treatment for brain cancer are at increased risk for other endocrine abnormalities, such as hypothyroidism [47], precocious puberty or hypogonadism. After 5 or more years from diagnosis, in CCSS-study one or more endocrine conditions were reported by 43% of childhood brain tumor survivors. Compared with siblings, survivors had a significantly increased risk of late-onset hypothyroidism [relative risk (RR) = 14.3], of growth hormone deficiency (RR = 277.8) and of the need for medications inducing puberty (RR = 86.1) [48]. Very few late effects were evident among those treated with surgery only, but risks were consistently elevated for those treated with radiation and surgery and higher for those who also received adjuvant chemotherapy [48]. It is useless to remember than typical symptoms of hypothyroidism are weariness and weight gain. Hypocortisolism resulting from insufficient substitution of the hypothalamic-pituitary-adrenal axis reduces physical activity. Overdosing with hydrocortisone, low levels of testosterone and low levels of GH are all associated with fatigue, a condition that, when chronic, can lead to obesity. Moreover, also precocious puberty could alter fat deposition, especially in girls.

### Other risk factors

Another risk factor for BMI increase is younger age at diagnosis [18]. Some biological reasons can explain this finding. They include the continued brain growth and mielinization until 4 years of age and the increased vulnerability of the developing brain to ionizing radiation. The CCSS-study in 921 brain tumor survivors found that female brain tumor survivors had a 3-fold increased risk for being obese if the age at diagnosis was 9 years or less compared with those diagnosed at older ages [39]. Data on the role of the hydrocephalus requiring ventriculoperitoneal shunt placement in weight gain are still conflicting [18]. A low ponderal index at birth, suggestive of intrauterine growth retardation, together with a maternal BMI greater than 25 kg/m^2 ^have to be considered as other risk factors for the development of obesity in craniopharyngioma patients as well as in common obesity [9].

## Metabolic consequences

An increased risk of cardio- and cerebrovascular mortality was observed in a study of 60 patients treated for craniopharyngioma compared to the general population during a median follow-up time of 12 years [49]. All the factors identified as causes of developing hypothalamic obesity, especially hypopituitarism and radiotherapy can be interpreted as risk factors for cardiovascular disease (CVD) later in life. Nevertheless, there is only limited data on the development of CVD in long term survivors of childhood central nervous system (CNS) cancer. It has already been demonstrated convincingly that adult GHD subjects are at increased risk for CVD [50,51]. In a retrospective study of patients with hypopituitarism, the risk of cardiovascular mortality was twice than in age- and gender-matched healthy controls. In long-term survivors of childhood CNS tumors (70% cases of medulloblastoma) who received cranial irradiation exceeding 45 Gy, Heikens et al described an altered risk profile for CVD with evidence of elevated systolic blood pressure, increased waist-hip ratio and adverse lipid profile [52]. Moreover, patients with GHD due to cranial irradiation presented a more pronounced altered metabolic profile as well as an increased intima-media thickness (IMT) of the carotid artery bulb [52]. The abnormalities were particularly pronounced in untreated GHD patients [53,54]. Srinivasan and colleagues were the first authors documenting an increased risk of metabolic syndrome (MS) in children with GHD and hypothalamic obesity after craniopharyngioma surgery. In fact, 9 patients post-craniopharyngioma surgery presented a higher fasting level of triglycerides and a lower high density lipoprotein cholesterol to total cholesterol ratio compared to those of healthy age-, sex-, pubertal stage- and BMI-matched controls [19]. Recently, after 4.9 ± 3.0 years from the diagnosis of craniopharyngioma, MS was detected in 73.3% (10/15) of survivors and in 20% (3/15) of age-, gender-, pubertal stage- and BMI-matched controls [27]. Moreover, children and adolescents with hypothalamic obesity following craniopharyngioma resection exhibited significantly more features of MS compared controls if they had a greater degree of impaired glucose tolerance, higher TNF-alpha and free fatty acids levels. All of these factors are known to contribute to increased cardiovascular risk in adults.

## Conclusion

The comprehension of the natural history and aetiology of obesity in craniopharyngioma survivors as well as the identification of modifiable risk factors should facilitate preventive interventions in the future. In fact, the management of obesity and eating disorders remains difficult, especially in patients with hypothalamic lesions. Nowadays, the best recommendation is the early beginning of prophylactic and therapeutic interventions in patients at risk of obesity. They should focus on promotion of a reduction in sedentary behavior and increases in physical activity. The American Cancer Society has developed specific guidelines on diet, nutrition and cancer prevention for the maintenance of health in the general population as well as in survivors of cancer [55]. The suggested diet is characterized by five or more servings of fruits and vegetables per day, the choice of whole grain foods and a limited amount of red meat, foods high in fat content and alcoholic beverages. In addition, 30-60 minutes of moderate to vigorous exercise at least or more than five times per week should be performed. Such interventions should be helpful in obesity prevention, but could also have a wide range of additional benefits in the prevention or amelioration of other late effects of cancer treatment. A multidisciplinary approach has to be offered to patients soon after the diagnosis of childhood cancer, continue through long-term follow up and extent into adulthood.

## Abbreviations

AGRP: Agouti-Related Protein; ARC: Arcuate Nucleus; BMI: Body Mass Index; CCSS: Childhood Cancer Survivor Study; CSN: Central Nervous System; CVD: Cardiovascular Disease; FSIGT: Frequently Sampled Intravenous Glucose Tolerance Test; GH: Growth Hormone; GHD: Growth Hormone Deficiency; Gy: Gray; GLP-1: Glucagon-Like Peptide 1; HOMA: Homeostasis Model Insulin Resistance Index; IMT: Intima Media Thickness; MS: Metabolic Syndrome; NPY: Neuropeptide Y; OGTT: Oral Glucose Tolerance Test; POMC: Pro-Opio-Melanocyte; RR: Relative Risk; SD: Standard deviation; SDS: Standard Deviation Score; Α-MSH: Alpha-Melanocyte Stimulating Hormone.

## Competing interests

The authors declare that they have no competing interests.

## Authors' contributions

Both the authors contributed to the conception of the review and were involved in writing, revising and approving the final draft of the manuscript.

## References

[B1] Pui CH Robison LL Look AT Acute lymphoblastic leukaemia Lancet 2008 371 1030 1043 10.1016/S0140-6736(08)60457-2 18358930

[B2] Oeffinger KC Mertens AC Sklar CA Kawashima T Hudson MM Meadows AT Friedman DL Marina N Hobbie W Kadan-Lottick NS Schwartz CL Leisenring W Robison LL Childhood Cancer Survivor Study Chronic health conditions in adult survivors of childhood cancer N Engl J Med 2006 355 1572 82 10.1056/NEJMsa060185 17035650

[B3] Maj JA Krieger MD Bowen I Geffner ME Craniopharyngioma in childhood Adv Pediatr 2006 53 183 209 10.1016/j.yapd.2006.04.013 17089867

[B4] Haupt R Magnani C Pavanello M Caruso S Dama E Garrè ML Epidemiological aspects of craniopharyngioma J Pediatr Endocrinol Metab 2006 19 Suppl 1 289 93 16700303

[B5] Müller HL Childhood craniopharyngioma--current concepts in diagnosis, therapy and follow-up Nat Rev Endocrinol 2010 6 11 609 18 10.1038/nrendo.2010.168 20877295

[B6] Spoudeas HA Saran F Pizer B A multimodality approach to the treatment of craniopharyngiomas avoiding hypothalamic morbidity: a UK perspective Pediatr Endocrinol Metab 2006 19 Suppl 1 447 51 10.1515/jpem.2006.19.4.447 16700323

[B7] Lee M Korner J Review of physiology, clinical manifestations, and management of hypothalamic obesity in humans Pituitary 2009 12 2 87 95 10.1007/s11102-008-0096-4 18327643

[B8] Fournier A Pauli A Cécile JP Cousin J Decherf A Craniopharyngioma having the appearance of an isolated obesity J Sci Med Lille 1968 86 3 171 175 5743043

[B9] Müller HL Emser A Faldum A Bruhnken G Etavard-Gorris N Gebhardt U Oeverink R Kolb R Sörensen N Longitudinal study on growth and body mass index before and after diagnosis of childhood craniopharyngioma J Clin Endocrinol Metab 2004 89 3298 3305 10.1210/jc.2003-031751 15240606

[B10] Müller HL Bueb K Bartels U Roth C Harz K Graf N Korinthenberg R Bettendorf M Kühl J Gutjahr P Sörensen N Calaminus G Obesity after childhood craniopharyngioma: German multicenter study on pre-operative risk factors and quality of life Klin Padiatr 2001 213 244 249 10.1055/s-2001-16855 11528558

[B11] Curtis J Daneman D Hoffman HJ The endocrine outcome after surgical removal of craniopharyngiomas Pediatr Neurosurg 1994 21 S1 24 27 7841075 10.1159/000120858

[B12] Crom DB Smith D Xiong Z Onar A Hudson MM Merchant TE Health status in long-term survivors of pediatric craniopharyngiomas J Neurosci Nurs 2010 42 6 323 8 10.1097/JNN.0b013e3181f8a59d 21207770 PMC4895693

[B13] Müller HL Gebhardt U Etavard-Gorris N Korenke E Warmuth-Metz M Kolb R Sörensen N Calaminus G Prognosis and sequela in patients with childhood craniopharyngioma -- results of HIT-ENDO and update on KRANIOPHARYNGEOM 2000 Klin Padiatr 2004 216 6 343 8 10.1055/s-2004-832339 15565549

[B14] Vinchon M Weill J Delestret I Dhellemmes P Craniopharyngioma and hypothalamic obesity in children Childs Nerv Syst 2009 25 3 347 52 10.1007/s00381-008-0754-x 19057910

[B15] Ahmet A Blaser S Stephens D Guger S Rutkas JT Hamilton J Weight gain in craniopharyngioma--a model for hypothalamic obesity J Pediatr Endocrinol Metab 2006 19 121 127 10.1515/JPEM.2006.19.2.121 16562584

[B16] de Vile CJ Grant DB Hayward RD Kendall BE Neville BG Stanhope R Obesity in childhood craniopharyngioma: relation to post-operative hypothalamic damage shown by magnetic resonance imaging J Clin Endocrinol Metab 1996 81 7 2734 7 10.1210/jc.81.7.2734 8675604

[B17] Müller HL Faldum A Etavard-Gorris N Gebhardt U Oeverink R Kolb R Sörensen N Functional capacity, obesity and hypothalamic involvement: cross-sectional study on 212 patients with childhood craniopharyngioma Klin Padiatr 2003 215 6 310 4 14677094 10.1055/s-2003-45499

[B18] Lustig RH Post SR Srivannabon K Rose SR Danish RK Burghen GA Risk factors for the development of obesity in children surviving brain tumors J Clin Endocrinol Metab 2003 88 611 616 10.1210/jc.2002-021180 12574189

[B19] Srinivasan S Ogle GD Garnett SP Briody JN Features of the metabolic syndrome after childhood craniopharyngioma J Clin Endocrinol Metab 2004 89 81 86 10.1210/jc.2003-030442 14715831

[B20] Iughetti L China M Berri R Predieri B Pharmacological treatment of obesity in children and adolescents: present and future J Obes 2011 2011 928165 21197151 10.1155/2011/928165 PMC3010692

[B21] Ghamari-Langroudi M Cone RD Shining a light on energy homeostasis Cell Metab 2011 13 3 235 6 10.1016/j.cmet.2011.02.007 21356511 PMC3073614

[B22] Roth C Wilken B Hanefeld F Schröter W Leonhardt U Hyperphagia in children with craniopharyngioma is associated with hyperleptinaemia and a failure in the downregulation of appetite Eur J Endocrinol 1998 138 1 89 91 10.1530/eje.0.1380089 9461323

[B23] Brabant G Horn R Mayr B von zur Mühlen A Honegger J Buchfelder M Serum leptin levels following hypothalamic surgery Horm Metab Res 1996 28 728 731 10.1055/s-2007-979888 9013752

[B24] Powley TL Laughton W Neural pathways involved in the hypothalamic integration of autonomic responses Diabetologia 1981 20 378 387 10.1007/BF00254506 27942825

[B25] Roth CL Hunneman DH Gebhardt U Stoffel-Wagner B Reinehr T Müller HL Reduced sympathetic metabolites in urine of obese patients with craniopharyngioma Pediatr Res 2007 61 4 496 501 10.1203/pdr.0b013e3180332cd6 17515878

[B26] Coutant R Maurey H Rouleau S Mathieu E Mercier P Limal JM Le Bouil A Defect in epinephrine production in children with craniopharyngioma: functional or organic origin? J Clin Endocrinol Metab 2003 88 12 5969 597 10.1210/jc.2003-030552 14671198

[B27] Simoneau-Roy J O'Gorman C Pencharz P Adeli K Daneman D Hamilton J Insulin sensitivity and secretion in children and adolescents with hypothalamic obesity following treatment for craniopharyngioma J Clin Endocrinol (Oxf) 2010 72 3 364 70 10.1111/j.1365-2265.2009.03639.x 19486023

[B28] Trivin C Busiah K Mahlaoui N Recasens C Souberbielle JC Zerah M Sainte-Rose C Brauner R Childhood craniopharyngioma: greater hypothalamic involvement before surgery is associated with higher homeostasis model insulin resistance index BMC Pediat 2009 2, 9 24 10.1186/1471-2431-9-24 PMC2675525 19341477

[B29] Lustig RH Hinds PS Ringwald-Smith K Christensen RK Kaste SC Schreiber RE Rai SN Lensing SY Wu S Xiong X Octreotide therapy of pediatric hypothalamic obesity: a double-blind, placebo-controlled trial J Clin Endocrinol Metab 2003 88 2586 2592 10.1210/jc.2002-030003 12788859

[B30] Hillman JB Tong J Tschop M Ghrelin biology and its role in weight-related disorders Discov Med 2011 11 61 521 8 21712018

[B31] Holmer H Pozarek G Wirfält E Popovic V Ekman B Björk J Erfurth EM Reduced energy expenditure and impaired feeding-related signals but not high energy intake reinforces hypothalamic obesity in adults with childhood onset craniopharyngioma J Clin Endocrinol Metab 2010 95 12 5395 402 10.1210/jc.2010-0993 20826582

[B32] Goldstone AP Patterson M Kalingag N Ghatei MA Brynes AE Bloom SR Grossman AB Korbonits M Fasting and postprandial hyperghrelinemia in Prader-Willi syndrome is partially explained by hypoinsulinemia, and is not due to peptide YY3-36 deficiency or seen in hypothalamic obesity due to craniopharyngioma J Clin Endocrinol Metab 2005 90 2681 2690 10.1210/jc.2003-032209 15687345

[B33] Kanumakala S Greaves R Pedreira CC Donath S Warne GL Zacharin MR Harris M Fasting ghrelin levels are not elevated in children with hypothalamic obesity J Clin Endocrinol Metab 2005 90 5 2691 5 10.1210/jc.2004-2175 15769982

[B34] O'Gorman CS Simoneau-Roy J Pencharz Mb P Adeli K Hamilton J Delayed ghrelin suppression following oral glucose tolerance test in children and adolescents with hypothalamic injury secondary to craniopharyngioma compared with obese controls Int J Pediatr Obes 2011 6 3-4 285 8 10.3109/17477166.2010.519388 21050078

[B35] Harz KJ Müller HL Waldeck E Pudel V Roth C Obesity in Patients with Craniopharyngioma: Assessment of Food Intake and Movement Counts Indicating Physical Activity J Clin Endocrinol Metab 2003 88 5227 5231 10.1210/jc.2002-021797 14602754

[B36] Müller HL Handwerker G Wollny B Faldum A Sorensen N Melatonin secretion and increased daytime sleepiness in childhood craniopharyngioma J Clin Endocrinol Metab 2002 87 3993 3996 10.1210/jc.87.8.3993 12161549

[B37] Poretti A Grotzer MA Ribi K Schönle E Boltshauser E Outcome of craniopharyngioma in children: long-term complications and quality of life Dev Med Child Neurol 2004 46 4 220 9 15077699 10.1017/s0012162204000374

[B38] Vinchon M Weill J Delestret I Dhellemmes P Craniopharyngioma and hypothalamic obesity in children Childs Nerv Syst 2009 25 3 347 52 10.1007/s00381-008-0754-x 19057910

[B39] Gurney JG Ness KK Stovall M Wolden S Punyko JA Neglia JP Mertens AC Packer RJ Robison LL Sklar CA Final height and body mass index among adult survivors of childhood brain cancer: Childhood Cancer Survivor Study J Clin Endocrinol Metab 2003 88 4731 4739 10.1210/jc.2003-030784 14557448

[B40] Müller HL Heinrich M Bueb K Etavard-Gorris N Gebhardt U Kolb R Sörensen N Perioperative dexamethasone treatment in childhood craniopharyngioma--influence on short-term and long-term weight gain Exp Clin Endocrinol Diabetes 2003 111 6 330 4 14520598 10.1055/s-2003-42722

[B41] DeVile CJ Grant DB Hayward RD Stanhope R Growth and endocrine sequelae of craniopharyngioma Arch Dis Child 1996 75 2 108 14 10.1136/adc.75.2.108 8869189 PMC1511642

[B42] Gonc EN Yordam N Ozon A Alikasifoglu A Kandemir N Endocrinological outcome of different treatment options in children with craniopharyngioma: a retrospective analysis of 66 cases Pediatr Neurosurg 2004 40 3 112 9 10.1159/000079852 15367800

[B43] Schmiegelow M Lassen S Weber L Poulsen HS Hertz H Müller J Dosimetry and growth hormone deficiency following cranial irradiation of childhood brain tumors Med Pediatr Oncol 1999 33 564 571 10.1002/(SICI)1096-911X(199912)33:6<564::AID-MPO8>3.0.CO;2-N 10573581

[B44] Siviero-Miachon AA Spinola-castro AM Guerra-Junior G Detection of metabolic syndrome features among childhood cancer survivors: a target to prevent disease Vascular Health and Risk Management 2008 4 4 825 836 19065999 10.2147/vhrm.s2881 PMC2597761

[B45] Angelin B Olivecrona H Rudling M Growth hormone and low-density lipoproteins Acta Endocrinol 1993 128 S2 26 8 8342389

[B46] Johansson JO Fowelin J Landin K Bengtsson BA Growth hormone deficient adults are insulin resistant Metabolism 1995 44 1126 9 10.1016/0026-0495(95)90004-7 7666785

[B47] Livesey EA Brook CG Thyroid dysfunction after radiotherapy and chemotherapy of brain tumors Arch Dis Child 1989 64 593 595 10.1136/adc.64.4.593 2751333 PMC1791957

[B48] Gurney JG Kadan-Lottick NS Packer RJ Neglia JP Sklar CA Punyko JA Stovall M Yasui Y Nicholson HS Wolden S McNeil DE Mertens AC Robison LL Childhood Cancer Survivor Study. Endocrine and cardiovascular late effects among adult survivors of childhood brain tumors: Childhood Cancer Survivor Study Cancer 2003 97 3 663 673 10.1002/cncr.11095 12548609

[B49] Bülow B Attewell R Hagmar L Malmström P Nordström CH Erfurth EM Postoperative prognosis in craniopharyngioma with respect to cardiovascular mortality, survival, and tumor recurrence J Clin Endocrinol Metab 1998 83 11 3897 904 10.1210/jc.83.11.3897 9814465

[B50] Bengtson BA The consequences of growth hormone deficiency in adults Acta Endocrinol 1993 128 S2 2 5 8342387

[B51] Pietilä S Mäkipernaa A Sievänen H Koivisto AM Wigren T Lenko HL Obesity and metabolic changes are common in young childhood brain tumor survivors Pediatr Blood Cancer 2009 52 7 853 9 10.1002/pbc.21936 19165891

[B52] Heikens J Ubbink MC van der Pal HPJ Bakker PJM Fliers E Smilde TJ Kastelein JJ Trip MD Long Term Survivors of Childhood Brain Cancer Have an Increased Risk for Cardiovascular Disease Cancer 2000 88 9 2116 2121 10.1002/(SICI)1097-0142(20000501)88:9<2116::AID-CNCR18>3.0.CO;2-U 10813724

[B53] Winter RJ Thompson RG Green OC Serum cholesterol and triglycerides in children with growth hormone deficiency Metabolism 1979 28 1244 1249 10.1016/0026-0495(79)90138-0 514086

[B54] Schaefer GB Greger NG Fesmire JD Blackett PR Wilson DP Frindik JP Lipids and apolipoproteins in growth hormone-deficient children during treatment Metabolism 1994 43 12 1457 1461 10.1016/0026-0495(94)90001-9 7990696

[B55] http://www.dietandcancerreport.org

